# Effects of oral fenugreek-derived preparations on testosterone-related outcomes in adult men: a systematic review and meta-analysis of randomized placebo-controlled trials

**DOI:** 10.3389/fnut.2026.1903508

**Published:** 2026-08-19

**Authors:** Pengyu Yang, Zeyu Niu, Jianlong Guo, Tingwei Huang, Degui Chang, Xiaopeng Huang

**Affiliations:** Hospital of Chengdu University of Traditional Chinese Medicine, Chengdu, Sichuan, China

**Keywords:** fenugreek, *Trigonella foenum-graecum*, testosterone, free testosterone, men’s health, systematic review, *meta-analysis*

## Abstract

**Background:**

Fenugreek (*Trigonella foenum-graecum*)-derived preparations are widely marketed for male-health benefits related to testosterone, sexual function, body composition, and exercise performance, yet the certainty, target population, and clinical interpretability of the available randomized evidence remain unclear.

**Objective:**

To systematically evaluate the effects of oral fenugreek-derived preparations on testosterone-axis outcomes in adult men and to descriptively summarize related male-health outcomes, including sexual function, symptoms, body composition, exercise performance, and safety.

**Methods:**

We included randomized placebo-controlled trials in adult men that evaluated oral fenugreek-derived preparations. Two authors independently searched PubMed, Embase, the Cochrane Central Register of Controlled Trials, Web of Science, and Scopus from inception to May 11, 2026, and additionally searched ClinicalTrials.gov and the World Health Organization International Clinical Trials Registry Platform (WHO ICTRP) for unpublished or grey evidence. Total testosterone and free testosterone were the primary outcomes. Risk of bias was assessed with the Cochrane Risk of Bias 2 (RoB 2) tool, certainty of evidence was graded using the Grading of Recommendations Assessment, Development and Evaluation (GRADE) approach, and the main pooled estimates were synthesized as standardized mean differences (SMDs) using random-effects models with restricted maximum likelihood (REML) estimation.

**Results:**

Thirteen studies were included, of which eight reported primary testosterone-axis data and five provided related or contextual male-health evidence. Six studies contributed to the total-testosterone meta-analysis, which showed a small favorable association with fenugreek (SMD: 0.25, 95% CI: 0.02, 0.48; *p* = 0.037; I^2^ = 0%, *p* = 0.578). Five studies contributed to the free-testosterone meta-analysis, for which the pooled estimate was imprecise and heterogeneous (SMD: 0.08, 95% CI: −0.48, 0.63; *p* = 0.725; I^2^ = 66.7%, *p* = 0.023). Across all 13 included studies, 11 were judged as having some concerns and 2 as high risk of bias; none was judged as low risk overall. The certainty of evidence for both primary testosterone-axis outcomes was very low.

**Conclusion:**

Oral fenugreek-derived preparations may be associated with a small favorable standardized effect on total testosterone in adult men, but the evidence is very uncertain and does not demonstrate a stable free-testosterone benefit. Current evidence does not support clinical or commercial testosterone-boosting claims.

**Systematic review registration:**

PROSPERO registration number: CRD420261364708. https://www.crd.york.ac.uk/PROSPERO/view/CRD420261364708

## 1 Introduction

Fenugreek (*Trigonella foenum-graecum*) is a plant used in food, traditional medicine, and modern dietary supplements. Its seeds contain multiple bioactive constituents, including steroidal sapogenins, saponins, trigonelline, 4-hydroxyisoleucine, flavonoids, and polyphenols (1–3). Recent genetic and phytochemical studies have further shown that fenugreek seed color and genotype are associated with differences in 4-hydroxyisoleucine, diosgenin, chlorophyll, steroidal saponins, and related biochemical traits (4, 5). In the context of men’s health, fenugreek-derived preparations are frequently discussed in relation to functional foods, dietary supplements, male sexual health, and “testosterone-boosting” products, and they appear in systematic reviews of herbal products as well as narrative reviews of testosterone boosters (6–11). This real-world context gives the topic practical relevance, but it also heightens the risk of overinterpretation: biological plausibility, supplement-market language, and favorable signals from individual trials do not, in themselves, demonstrate that fenugreek can produce stable and clinically meaningful androgenic effects.

Testosterone-related outcomes are not homogeneous measures. Total testosterone, free testosterone, calculated free testosterone, free testosterone index, salivary testosterone, sex hormone-binding globulin (SHBG), and symptom or sexual-function scales are all influenced by assay platform, sampling time, binding-protein status, calculation formula, baseline population characteristics, and reporting practice (12–15). In clinical practice, the diagnosis of male hypogonadism cannot rely on a single isolated biochemical measurement; it requires corroborating symptoms, signs, and reliable repeat testing (16–18). Accordingly, in supplement research, treating mild changes in total testosterone, unstable free-testosterone findings, questionnaire improvements, body-composition changes, and performance gains as a single undifferentiated “male-health benefit” risks overstating the strength of the evidence and misattributing non-testosterone-mediated findings to testosterone-axis effects.

Previous reviews have examined fenugreek, herbal products, and testosterone-related outcomes from several angles. The closest prior meta-analysis focused on fenugreek extract and testosterone levels in men, while other reviews evaluated broader herbal effects on male testosterone concentrations, phytotherapeutic interventions for male reproductive or prostate-related parameters, and the relationship between fenugreek and anabolic or performance outcomes (10, 19–21). These reviews confirm ongoing interest in the topic, but they also reveal inconsistency in search dates, eligible populations, formulation definitions, handling of coformulations and special settings, outcome classification, and certainty interpretation. When recent randomized trials, coformulations or mixed extracts, acute crossover studies, exercise cointerventions, and disease-specific populations coexist within the same evidence base, broadening inclusion alone does not automatically strengthen the credibility of the conclusions. More important is identifying which studies genuinely contribute to the primary pooled testosterone-axis evidence and which can only be interpreted as related male-health or contextual evidence.

This systematic review and meta-analysis therefore aimed to update and hierarchically evaluate randomized placebo-controlled trial evidence on oral fenugreek-derived preparations in adult men. We treated the testosterone axis as the primary evidence domain, with total testosterone and free testosterone as the main outcomes, while retaining sexual-function questionnaires, Aging Males’ Symptoms (AMS), body composition, exercise performance, and safety biomarkers as related and supplementary outcomes. This design was intended to address two questions: whether fenugreek shows a quantifiable and interpretable testosterone-axis effect, and whether any such effect is sufficient to support broader male-health, exercise-performance, or commercial testosterone-boosting claims.

## 2 Methods

### 2.1 Reporting standards and registration

The design, conduct, and reporting of this review followed the Preferred Reporting Items for Systematic Reviews and Meta-Analyses (PRISMA) 2020 statement and its explanation and elaboration documents (22, 23), as well as the requirements of A MeaSurement Tool to Assess systematic Reviews 2 (AMSTAR 2) (24), to support methodological rigor and reporting completeness. The review protocol was registered in the International Prospective Register of Systematic Reviews (PROSPERO; CRD420261364708).

### 2.2 Information sources and search strategy

We systematically searched PubMed, Embase, the Cochrane Central Register of Controlled Trials (CENTRAL), Web of Science, Scopus, ClinicalTrials.gov, and the WHO International Clinical Trials Registry Platform (WHO ICTRP) from inception to May 11, 2026, without language restriction. From CENTRAL, only records listed under the Trials tab were imported; Cochrane Reviews were not imported as trial records. ClinicalTrials.gov API v2 results were retained as the primary registry source and were used together with manually downloaded registration records to identify potentially unpublished or ongoing evidence. The main PubMed strategy combined intervention terms—fenugreek, *Trigonella*, and specific extract names—with trial-design terms such as randomized, placebo, controlled, crossover, and double-blind; focused supplementary searches targeting the testosterone axis and prior evidence syntheses were also conducted. Full search strategies, run dates, and record counts are provided in Supplementary Table S1.

### 2.3 Eligibility criteria

Reports were eligible for the systematic review if they met all of the following criteria: participants were adult men (aged ≥18 years); the study design was a randomized placebo-controlled trial; the intervention was an oral fenugreek-derived or fenugreek-containing preparation; and the report included at least one testosterone-axis biomarker or a prespecified related male-health outcome (with total testosterone and free testosterone as the main outcomes, and sexual-function questionnaires, AMS, body composition, exercise performance, and safety biomarkers treated as related or supplementary outcomes). Among studies meeting these criteria, chronic parallel-group randomized controlled trials (RCTs) using separable fenugreek-derived preparations and reporting testosterone-axis biomarkers were prespecified as the primary testosterone-axis evidence layer and contributed to the total-testosterone or free-testosterone meta-analysis according to the specific outcome reported.

Exclusion criteria encompassed: non-human or *in vitro* studies; non-randomized studies, observational studies, case reports, reviews, commentaries, and mechanistic or pharmacological experiments; intervention studies without a placebo or control group; studies not conducted in adult men or those in which adult-male data could not be separated; interventions that did not contain fenugreek, were not administered orally, or did not allow confirmation of fenugreek exposure; studies that did not report any testosterone-axis biomarker or prespecified related male-health outcome; conference abstracts, registration records, protocols, or news/product materials without extractable outcome data; and secondary reports duplicating an already included original trial. Duplicate reports were linked to the primary trial and were not counted as independent study units. Registry and observation-list records were retained for background tracking only and were not counted as published randomized trials.

### 2.4 Study selection and data extraction

Two reviewers (P.Y.Y and Z.Y.N.) independently screened titles and abstracts, assessed full-text eligibility, and extracted data using a predefined standardized form. Disagreements were resolved by consensus. Extracted information included first author, publication year, country, study design, participant characteristics (number of participants per group and health status), intervention characteristics (dose, preparation type, and duration), outcomes, adverse events, and funding information.

For continuous outcomes reported as medians with interquartile ranges or ranges rather than means and standard deviations (SDs), we used established methods to estimate approximate means and SDs for inclusion in quantitative synthesis (25, 26). In multidose studies sharing the same placebo group, only one prespecified primary comparison was retained in the main pooled analysis to avoid duplicate counting of the shared control group. All extracted results were cross-checked by two investigators (P.Y.Y. and Z.Y.N.), and disagreements were resolved through discussion.

### 2.5 Study classification and evidence-synthesis framework

Included studies were classified according to study design and reporting characteristics as follows. Chronic parallel-group RCTs reporting total testosterone or free testosterone data were included in the main quantitative meta-analysis of testosterone-axis outcomes. Studies meeting the general review eligibility criteria but unsuitable for the main testosterone-axis pooled analysis—because of acute single-dose crossover design, inseparable coformulations, mixed botanical extracts, symptom-only reporting without hormone-level data, or performance/safety outcomes without testosterone-axis endpoints—were retained as supportive or contextual studies. These studies were not quantitatively pooled as primary testosterone-axis evidence, but were used for qualitative summaries, supplementary quantitative analyses, or contextual interpretation of related outcomes such as sexual-function questionnaires, AMS, body composition, exercise performance, and safety biomarkers.

Repeated publications or secondary abstract reports from the same trial were linked to the original trial and were not counted as independent studies. Clinical trial registry and observation-list records were used only to track potential evidence and to inform background interpretation of publication bias; they were not counted as published randomized trials. For issues such as unit conversion, conversion of the standard error of the mean (SEM) to SD, graphical reconstruction, unclear denominators, disease-specific populations, and exercise or athlete settings, standard formulas were applied for data conversion and the potential influence of these issues was examined through sensitivity analyses within the above classification framework.

### 2.6 Risk of bias and certainty assessment

Risk of bias was assessed using the Cochrane RoB 2 tool (27). The assessed domains included the randomization process, deviations from intended interventions, missing outcome data, measurement of the outcome, and selection of the reported result. Two reviewers (P.Y.Y and J.L.G.) independently assessed each domain, with ratings of low risk, some concerns, or high risk, and disagreements were resolved through discussion (D.G.C.). The certainty of evidence for the two main pooled testosterone-axis outcomes was assessed using the GRADE approach (28–30). Risk of bias, inconsistency, indirectness, imprecision, and publication bias were explicitly considered in outcome-level judgments (31, 32). Because testosterone outcomes were synthesized as SMDs across studies using different assay units and populations, the GRADE interpretation did not convert SMDs into absolute testosterone changes and did not treat small standardized effects as automatically equivalent to clinically meaningful changes.

### 2.7 Statistical analysis

Because total testosterone and free testosterone were reported using different assay methods and units across studies (33, 34), the main testosterone-axis outcomes were synthesized as SMDs using Hedges’ g for small-sample correction, together with 95% confidence intervals (95% CIs), in random-effects models with REML estimation of between-study variance. Related and supplementary outcomes with aligned units were synthesized as mean differences (MDs); where needed, generic inverse-variance methods were used for outcomes such as the Derogatis Interview for Sexual Function (DISF) total score. Heterogeneity was described using I^2^, tau^2^, and Q-test results, and was interpreted in conjunction with study classification, formulation, population, and reporting characteristics (35, 36).

For the main testosterone-axis syntheses, leave-one-out sensitivity analyses were performed to assess robustness. Prespecified deletion sensitivity analyses examined exclusion of studies in benign prostatic hyperplasia (BPH) populations, exercise or athlete settings, product/formulation-boundary studies, high-risk dose-boundary comparisons, and, where applicable, rows requiring SEM-to-SD conversion. For outcomes with at least 10 contributing studies, potential publication bias would be assessed using funnel plots, Begg’s test, and Egger’s test, with trim-and-fill used if indicated. Conditional subgroup analyses, dose–response analyses, meta-regression, and trial sequential analysis were not included in the main manuscript. All analyses were performed in R version 4.5.3 using metafor version 4.8–0. A two-sided *p* value < 0.05 was considered statistically significant.

## 3 Results

### 3.1 Study selection

**Figure 1:**
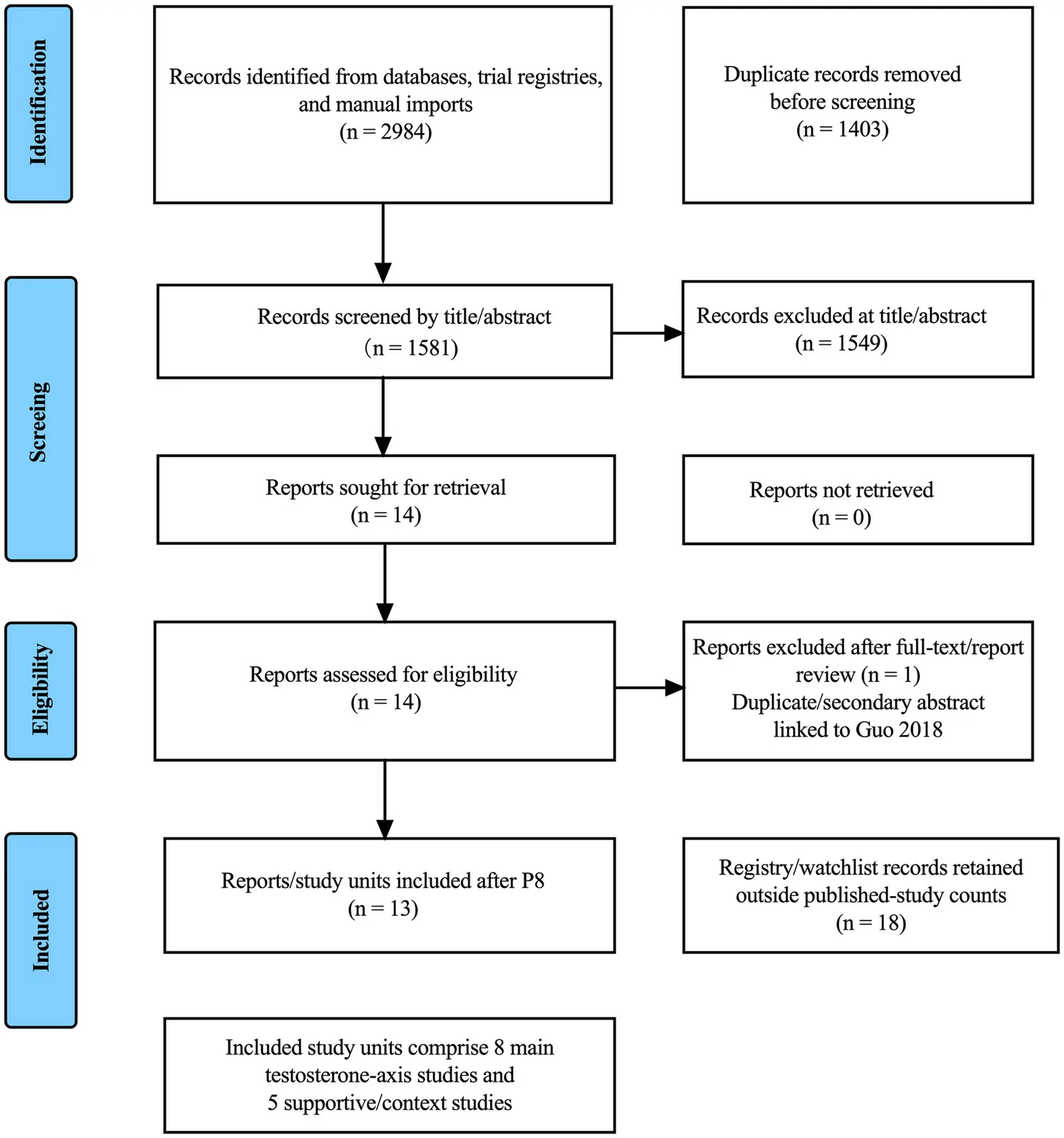
Flowchart of the literature screening process.

A total of 2,984 records were identified. After preliminary deduplication based on DOI, PMID, registration number, and title, 1,403 duplicate records were removed, leaving 1,581 records for title and abstract screening. Of these, 1,549 clearly irrelevant records were excluded, and 14 potentially relevant full-text reports were subsequently assessed for eligibility. One duplicate or secondary abstract report related to the Guo 2018 Furosap trial was excluded, leaving 13 included studies. Of these, eight reported primary testosterone-axis data and five provided related male-health, acute, mixed-formulation, or performance/safety evidence. Eighteen registry or observation-list records were retained solely to track potentially unpublished or ongoing evidence and were not counted in the denominator of published randomized trials or quantitative synthesis (Supplementary Table S2). The PRISMA flow diagram is shown in Figure 1.

### 3.2 Study characteristics

Thirteen studies met the inclusion criteria, and their key characteristics are summarized in Table 1. All included studies were randomized placebo-controlled trials. According to the prespecified evidence-synthesis framework, eight were classified as primary testosterone-axis evidence (all chronic parallel-group RCTs), whereas the remaining five were treated as supportive or contextual studies for qualitative summary or background interpretation.

**Table 1:** Study characteristics of the included trials.

Study unit	Country	Design	Participants, n (I/C)	Population	Intervention	Dose and duration	Control	Role in review	Key outcomes/contribution	Funding/notes
Wilborn 2010 (37)	USA	Double-blind, placebo-controlled, parallel-group randomized controlled trial	30 Analyzed (17/13)	Resistance-trained men	*Trigonella foenum-graecum* standardized for Grecunin (fenugreek extract), capsule	500 mg/day, 8 weeks	Maltodextrin placebo	Main testosterone-axis, boundary exercise/product study	Total testosterone; bioavailable testosterone; body composition; strength	Indus Biotech; exercise-trained/product boundary
Poole 2010 (38)	USA	Double-blind, placebo-controlled, parallel-group trial	49 Analyzed (26/23)	Resistance-trained males	Fenugreek product standardized for 70% TRIGIMANNOSE, capsules	500 mg/day, 8 weeks	Placebo capsules	Main testosterone-axis, outcome-specific boundary study	Free testosterone; strength; body fat; no total-testosterone endpoint	Indus Biotech; exercise-trained/product boundary
Wankhede 2016 (39)	India	Randomized controlled trial	60 Randomized; 55 PP (29/26)	Healthy male subjects	Fenugreek glycoside fraction (Fenu-FG), capsule	600 mg/day, 8 weeks	Matching placebo	Main testosterone-axis, exercise-training boundary study	Total testosterone; free testosterone; body fat; strength	Indus Biotech Private Ltd.; pilot/industry-affiliated
Rao 2016 (40) Testofen	Australia	Double-blind randomized placebo-controlled trial	120 Randomized; 111 completed (56/55); hormones analyzed 97 (47/50)	Healthy aging males	Testofen (*Trigonella foenum-graecum* seed extract), standardized seed extract tablets	600 mg/day, 12 weeks	Placebo	Main testosterone-axis core anchor	Total testosterone; calculated free testosterone; SHBG; prolactin; AMS; DISF; grip strength	Gencor Pacific, Hong Kong; sexual-function supportive anchor
Guo 2018 (41) Furosap	USA	Randomized, double-blind, placebo-controlled trial	40 Randomized; 35 completed (I/C NR)	Healthy male athletes	Furosap, fenugreek seed extract enriched in 20% protodioscin, oral capsules	500 mg/day, 12 weeks	Placebo capsules	Main testosterone-axis, athlete/product boundary study	Total testosterone; lean/fat-free mass; exercise performance	Industry-funded athlete/product boundary; duplicate abstract D00415 linked as a secondary report
Rao-Mallard-Grant 2020 (42)	Australia	Double-blind randomized placebo-controlled trial	138 Randomized; 98 completed (31/35/32; 600/300/C); hormones analyzed 78 (23/29/26)	Healthy exercising males	Testofen (fenugreek extract), capsule	600 mg/day selected comparison, 8 weeks	Maltodextrin placebo	Main testosterone-axis, dose-arm/exercise boundary study	Total testosterone; free testosterone; SHBG; strength; body composition	Gencor Pacific Ltd.; shared placebo and dose-arm rule required
Rao 2020 (43) BPH	Australia	Double-blind randomized placebo-controlled trial	100 Randomized; 84 completed (42/42)	Men with benign prostatic hyperplasia	*Trigonella foenum-graecum* extract (Testofen), oral capsules	600 mg/day, 12 weeks	Maltodextrin placebo	Main testosterone-axis, BPH boundary study	Total testosterone; free testosterone; SHBG; IPSS; PSA	Gencor Pacific Limited; disease-specific boundary
Lee-Ødegård 2024 (44)	Norway	Double-blind randomized controlled trial	100 Enrolled; 95 completed (21/25/27/22; 600/1200/1800/C)	Men aged 40–80 years	Trigozim fenugreek extract, oral tablets	600/1200/1800 mg/day, 12 weeks	Placebo with vitamins and minerals	Main testosterone-axis core/multi-dose study	Plasma total testosterone; free testosterone index; saliva testosterone; SHBG; libido/safety	PurOmega Ltd.; matched micronutrient placebo matrix; multi-dose boundary
Steels 2011 (45)	Australia	Double-blind randomized placebo-controlled trial	60 Randomized; 54 completed (27/27)	Healthy males without erectile dysfunction or low libido	Maxinbed, standardized *Trigonella foenum-graecum* extract with magnesium, zinc, and pyridoxine, oral tablet	600 mg/day, 6 weeks	Placebo	Supportive sexual-function/coformulation study	Sexual-function questionnaires; testosterone context; prolactin/PSA safety	Coformulation boundary; supportive sexual-function context
Mokashi 2014 (46) IND9	India	Randomized, double-blind, placebo-controlled, two-period crossover trial	16 Randomized (8/8 sequence groups)	Healthy sedentary male subjects	Glycosides-based standardized fenugreek seed extract (IND9), capsules	600 mg single-dose/acute exposure; chronic duration not applicable	Matching placebo	Supportive acute crossover/special-case study	Acute testosterone-axis biomarkers; acute safety	Indus Biotech Private Limited; not mixed with chronic parallel RCT main pool
Park 2018 (47) TFGL	South Korea	Randomized, double-blind, placebo-controlled clinical trial	88 Randomized/completed (44/44)	Men aged >40 years with testosterone deficiency syndrome symptoms	Mixed *Trigonella foenum-graecum* seed + *Lespedeza cuneata* extract (TFGL), capsules	400 mg/day, 8 weeks	Matching placebo capsules	Supportive mixed-extract/context study	Total testosterone; free testosterone; AMS/ADAM/IIEF; body composition; lipids; safety	Mixed extract not separable from Lespedeza; contextual supportive row rather than fenugreek-alone mainline evidence
Hausenblas 2020 (48)	USA	Randomized double-blind placebo-controlled parallel trial	57 Randomized; 56 analyzed (19/19/18; 400/500/C)	Healthy recreationally active men	Fenugreek seed extract (AlphaFen), liposomal/lipid carrier capsule	400 or 500 mg/day, about 8.6 weeks	Placebo	Supportive symptom-only report; hormones collected but not reported in this report	AMS domains; quality of life; safety; no hormone outcome in report	SPECNOVA; track possible companion hormone report
Thakurdesai 2024 (49) FEDE	Australia	Randomized, double-blind, placebo-controlled, three-arm parallel trial	153 Randomized; 99 completed (arm counts NR; 300/600/C)	Recreationally active young male subjects	FEDE (Enducor) fenugreek-derived standardized extract, oral capsules	300 or 600 mg/day, 8 weeks	Matching maltodextrin placebo capsules	Supportive performance/safety context study	Endurance performance; VO2max/MET; fatigue; safety labs; no testosterone-axis outcome	Supportive performance/safety context; no testosterone-axis outcome for the main synthesis

AMS, Aging Males’ Symptoms; ADAM, Androgen Deficiency in the Aging Male; BPH, benign prostatic hyperplasia; DISF, Derogatis Interview for Sexual Function; IPSS, International Prostate Symptom Score; PSA, prostate-specific antigen; SHBG, sex hormone-binding globulin; TFGL, *Trigonella foenum-graecum* + *Lespedeza cuneata* mixed extract; VO2max, maximal oxygen uptake; MET, metabolic equivalent. Main testosterone-axis study units are listed before supportive/context study units for reader-facing orientation. Participant values follow the reporting basis available in each source: enrolled = entered the trial, randomized = allocated, completed = completed follow-up, PP = per-protocol, and analyzed = reported or extracted analysis denominator. I/C = intervention/control; C = control/placebo; NR = not reported or not reliably extractable. For two-arm parallel trials, values in parentheses are intervention/control counts; for multi-arm trials, counts are expanded by dose labels (for example, 600/300/C). When arm-specific completed or analyzed counts were not extractable, or when denominators differed by outcome, study-level totals are shown in table and outcome-specific denominators are retained in the analysis dataset. In crossover studies, parenthetical counts may indicate randomization sequence groups rather than independent parallel arms. Park 2018 TFGL used a mixed Trigonella + Lespedeza product and is therefore retained as contextual supportive evidence rather than fenugreek-alone mainline evidence. Steels 2011 was a coformulation study, and Mokashi 2014 IND9 was an acute crossover study; these rows should not be interpreted as directly parallel to the chronic main testosterone-axis RCTs. Hausenblas 2020 and Thakurdesai 2024 FEDE contribute supportive symptom/performance or safety context and are not main testosterone-axis pooled studies. Bagchi/Swaroop 2023 is a duplicate/secondary abstract linked to Guo 2018 and is not counted as an independent study unit in this table.

The eight primary testosterone-axis studies involved 637 randomized participants and included resistance-trained men, healthy middle-aged or older men, healthy male athletes, recreationally active men undergoing calisthenics training, patients with BPH, and a multidose intervention trial in men aged 40–80 years (37–44). Most interventions used oral fenugreek-derived preparations at daily doses of 500–600 mg over 8–12 weeks. Lee-Ødegård et al. evaluated three dose levels of 600, 1,200, and 1,800 mg/day (44). There was clear clinical and methodological heterogeneity across the primary testosterone-axis study units. Four studies involved exercise training or performance-related cointerventions (37–39, 42). One study (Guo 2018) used a proprietary extract (41), and one study (Rao 2020) specifically enrolled a BPH population (43). Two additional studies provided relatively direct testosterone-axis evidence (40, 44). Consequently, although 13 studies were included in the systematic review, only six contributed to the total-testosterone analysis and five to the free-testosterone analysis. The remaining five supportive or contextual studies were retained to preserve the broader male-health, acute-effect, mixed-formulation, and performance-related context, but were not treated as primary testosterone-axis pooled evidence (45–49). Industry funding or company involvement was common across the included studies. Eight of the 13 studies provided some safety-related information, ranging from adverse-event statements to PSA, prolactin, or routine laboratory monitoring. Among studies that reported serious-adverse-event status, no serious adverse events were reported; however, adverse-event denominators, event definitions, and collection methods were often unclear, and long-term safety remains uncertain (Supplementary Table S3).

### 3.3 Risk of bias

**Figure 2:**
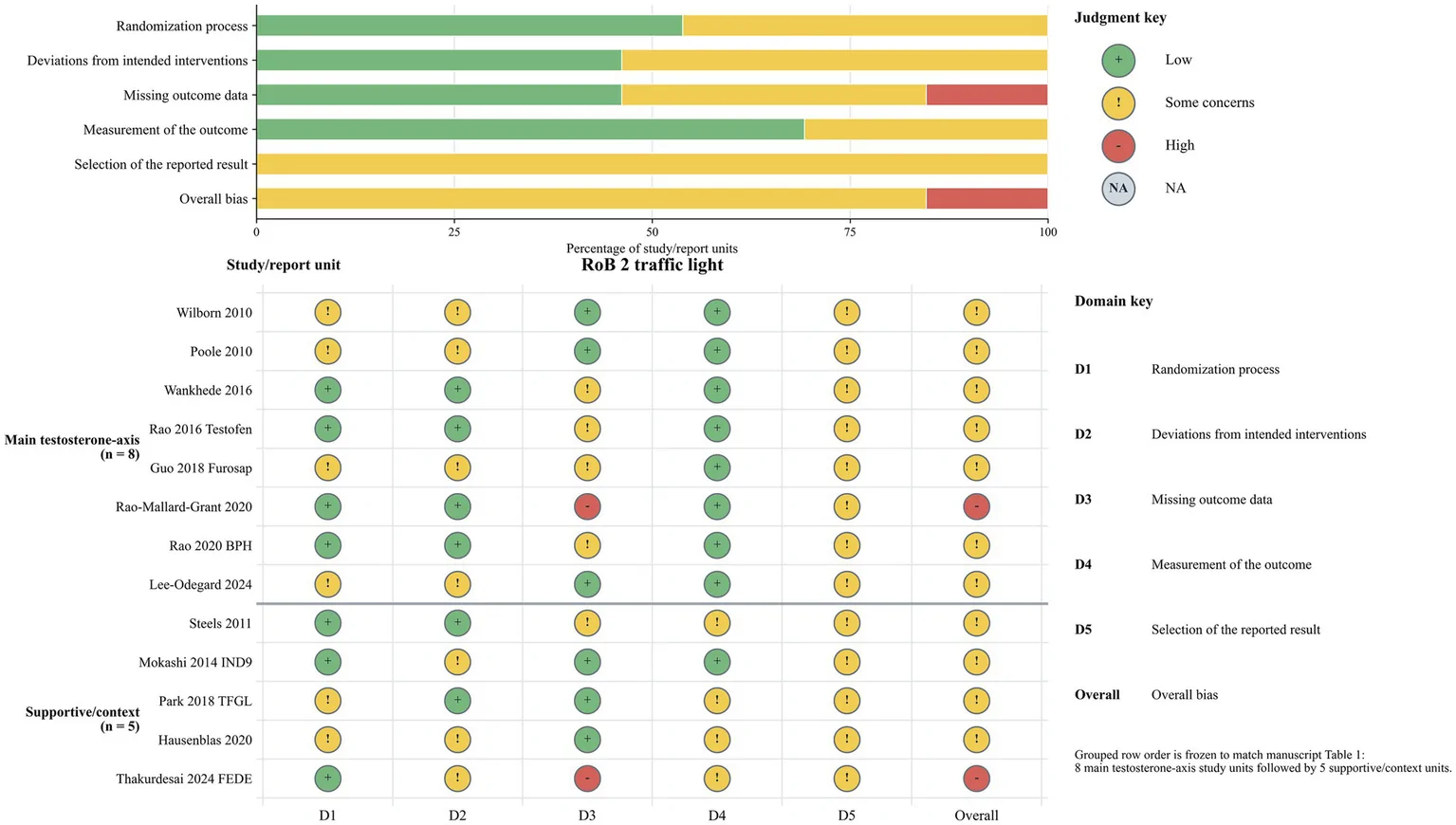
Visualization and traffic light plots of the risk of bias assessment using RoB 2.

Risk of bias was assessed for all 13 included studies using the Cochrane RoB 2 tool (Figure 2). Overall, 11 studies (84.6%) were judged as having some concerns and 2 (15.4%) as having high risk of bias; no study was judged as low risk overall. Among the eight primary testosterone-axis studies, seven (87.5%) were rated as having some concerns and one (12.5%) as high risk. Among the five related or contextual study units, four were rated as having some concerns and one as high risk. The most common source of downgrading was selection of the reported result, primarily because of limited protocol or registry traceability, multiple outcome domains, reporting across multiple time points or measures, or insufficient information to confirm that all expected outcomes were fully reported. Missing outcome data were also an important concern, particularly in Rao-Mallard-Grant 2020 and Thakurdesai 2024 FEDE. For testosterone-axis outcomes, objective hormone measurements reduced some concerns about outcome measurement, but incomplete reporting of hormone-analysis denominators, completer populations, or per-protocol handling still reduced confidence in the estimates.

### 3.4 Certainty of evidence

The certainty of evidence for the two primary testosterone-axis outcomes was assessed using GRADE. Both total testosterone and free testosterone were rated as very low certainty. Table 2 presents the corresponding GRADE evidence profile. Total testosterone was downgraded for risk of bias, indirectness, imprecision, and suspected publication bias. Indirectness reflected the mixture of healthy or exercise-trained men, patients with BPH, proprietary preparations, dose-boundary comparisons, and exercise settings. Imprecision reflected the small effect size and the limited clinical interpretability of the result given the modest sample size. Because fewer than 10 studies contributed to the pooled estimate, no formal funnel-plot or publication-bias test was performed; however, industry sponsorship and the small-study setting justified maintaining concern about potential publication bias.

**Table 2:** GRADE summary of findings for the main testosterone-axis outcomes.

Outcomes	Risk of bias	Inconsistency	Indirectness	Imprecision	Publication bias	Quality of evidence
Total testosterone	Serious	Not serious	Serious	Serious	Serious, suspected	⊕○○○ Very low
Free testosterone	Serious	Serious	Serious	Serious	Serious, suspected	⊕○○○ Very low

Total testosterone included 6 studies and 385 comparison participants; pooled effect SMD 0.25 (95% CI 0.02 to 0.48), I^2^ = 0%. Free testosterone included 5 studies and 334 comparison participants; pooled effect SMD 0.08 (95% CI -0.48 to 0.63), I^2^ = 66.7%. No pooled testosterone-axis outcome was supported only by low-risk studies; Rao-Mallard-Grant 2020 was high overall risk and the remaining pooled studies were mostly rated as Some concerns. Indirectness was serious for both outcomes because the evidence mixed healthy or exercise-trained men, BPH populations, proprietary products or formulations, and dose-arm boundaries. Publication bias was suspected for both outcomes. Formal funnel or publication-bias testing was not run because *k* < 10; interpretation should consider the sponsor and industry context. Absolute effects were not calculated because the syntheses used standardized mean differences across mixed testosterone assay units.

### 3.5 Main testosterone-axis outcomes

**Figure 3:**
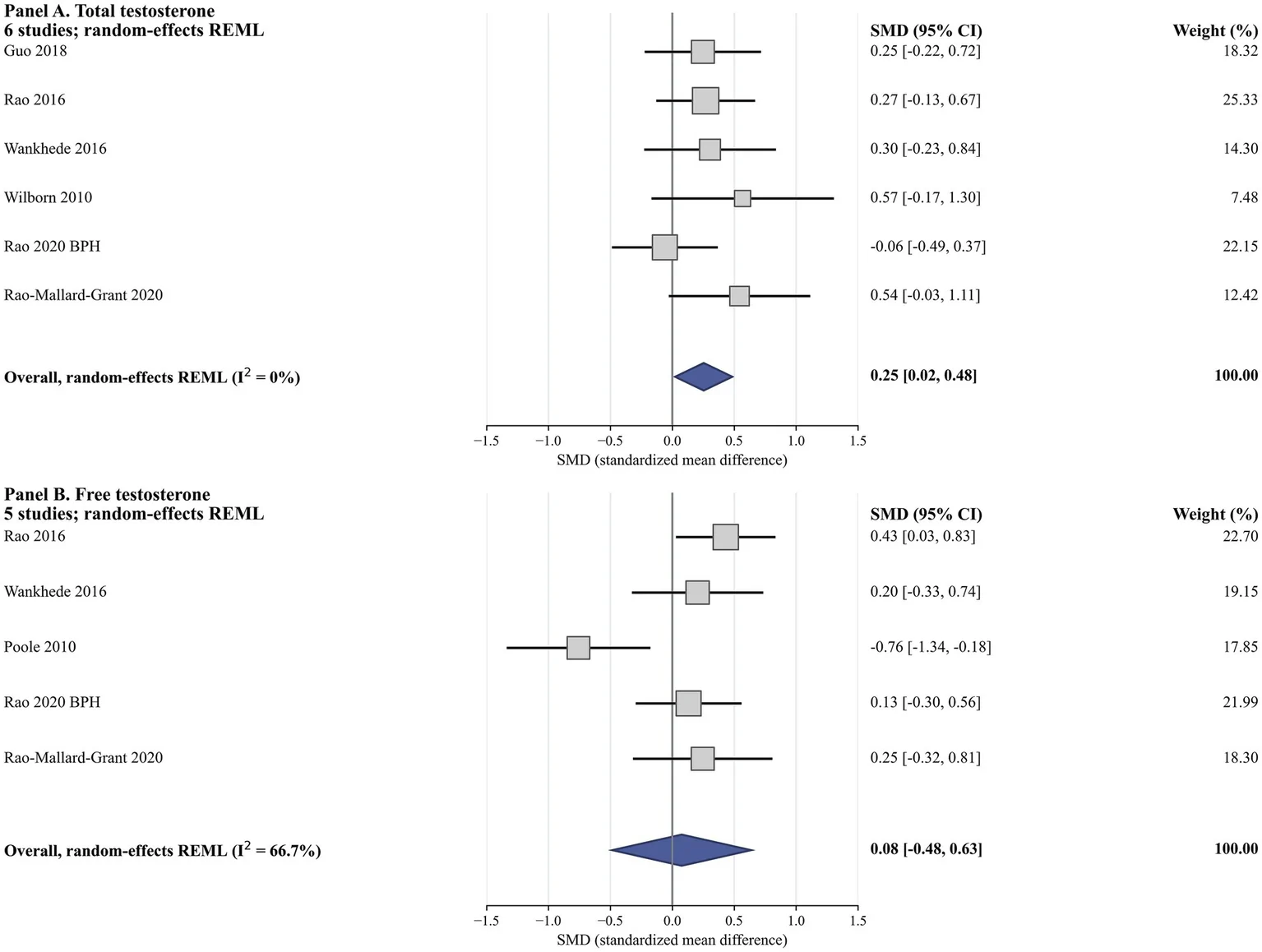
Forest plots of testosterone-axis outcomes: **(A)** total testosterone; **(B)** free testosterone. Effect sizes are expressed as standardized mean differences (SMDs). Source-note caveats carried into the figure note: Poole 2010 free-testosterone unit-label trace; Rao 2020 BPH free-testosterone unit traced from pathology-table context; Guo 2018 total-testosterone SEM-to-SD conversion; Rao 2020 calisthenics prose unit typo did not override the Table 4 nmol/L unit. The Rao 2020 calisthenics hormone display uses only the locked 600 mg comparison because the 300 mg arms share the placebo group.

Six studies contributed to the primary pooled analysis of total testosterone, comprising 385 participants in total (193 in the fenugreek groups and 192 in the placebo groups). Random-effects meta-analysis showed that oral fenugreek-derived preparations were associated with a small increase in total testosterone (SMD: 0.25, 95% CI: 0.02, 0.48; *p* = 0.037; I^2^ = 0%, *p* = 0.578; Figure 3A). The direction of effect favored fenugreek, but the magnitude was small and, because SMD was used, the result cannot be directly interpreted as an absolute increase in serum testosterone in any single unit.

Five studies contributed to the primary pooled analysis of free testosterone, representing 334 participants in total (167 in the fenugreek groups and 167 in the placebo groups). The pooled estimate for free testosterone crossed the null and showed substantial heterogeneity (SMD: 0.08, 95% CI: −0.48, 0.63; *p* = 0.725; I^2^ = 66.7%, *p* = 0.023; Figure 3B). The free-testosterone analysis therefore does not support a stable pooled benefit and cannot serve as biological confirmation of the small increase observed for total testosterone.

### 3.6 Sensitivity analyses and robustness of findings

Prespecified deletion and leave-one-out sensitivity analyses were performed to assess the robustness of the main outcomes. For total testosterone, the overall direction of effect remained favorable after exclusion of individual studies, suggesting some directional stability, although the magnitude and precision of the pooled estimate were influenced by specific studies. Excluding the BPH study increased the pooled effect to SMD: 0.34 (95% CI: 0.17, 0.52). By contrast, excluding product/formulation-boundary studies, excluding the Rao-Mallard-Grant 2020 dose-boundary comparison, or excluding rows requiring SEM-to-SD conversion reduced precision and shifted the confidence interval toward the null (Supplementary Table S5).

For free testosterone, exclusion of individual studies likewise did not materially change the overall direction of effect. Excluding the BPH study or the Rao-Mallard-Grant 2020 dose-boundary comparison still yielded imprecise estimates that crossed the null. Excluding product/formulation-boundary studies shifted the estimate in a favorable direction (SMD: 0.27, 95% CI: 0.04, 0.49) (Supplementary Table S5), but this result was observed only under a specific exclusion condition and should therefore be interpreted cautiously as a boundary finding rather than as a change in the main conclusion. Overall, the sensitivity analyses support the main interpretation: total testosterone showed a small and directionally stable positive effect, whereas free testosterone remained weak and unstable.

These sensitivity-analysis patterns were also consistent with the characteristics of the individual studies. Rao et al., conducted in a BPH population, did not show improvement in total testosterone, free testosterone, or SHBG; excluding this study strengthened the total-testosterone estimate, suggesting that baseline population characteristics and indication boundaries may influence the pooled result (43). By contrast, both Poole et al. and Wankhede et al. were conducted in resistance-training contexts but did not show consistent directions or magnitudes of effect for free testosterone, indicating that performance or body-composition improvement should not automatically be interpreted as evidence of stable free-testosterone elevation (38, 39). The multidose design and exercise background of Rao et al. likewise suggest that selection of a single dose arm can influence precision and generalizability (42).

### 3.7 Related and supplementary quantitative findings

For related male-health outcomes, pooled analyses favored fenugreek for the AMS psychology domain (MD: −1.31, 95% CI: −2.37, −0.25), AMS somatic domain (MD: −2.22, 95% CI: −3.52, −0.91), AMS total score (MD: −4.56, 95% CI: −7.98, −1.15), and DISF total score (MD:16.97, 95% CI: 6.06, 27.88). The pooled AMS sexual domain remained imprecise (MD: −0.95, 95% CI: −2.45, 0.55) (Supplementary Table S4). These findings suggest possible favorable directions for some symptom and sexual-function questionnaires, but because they were not primary testosterone-axis outcomes, involved small numbers of studies, and in some cases were affected by formulation or design boundaries, they cannot be used to infer a clear testosterone-mediated clinical effect.

Among supplementary outcomes, body-fat percentage (MD: −1.37 percentage points, 95% CI: −2.13, −0.61) and bench-press one-repetition maximum (1RM) (MD: 4.86 kg, 95% CI: 1.71, 8.02) favored fenugreek. SHBG, fat-free mass, prolactin, and leg-press 1RM did not show stable findings, primarily because the pooled estimates were imprecise, heterogeneous, or both. Only directionally relevant summaries are presented in the main manuscript; full quantitative results are provided in Supplementary Table S4.

Several individual studies are particularly informative for interpreting these related and supplementary findings. Rao et al. Testofen reported improvements in AMS, DISF, total testosterone, and calculated free testosterone, making it one of the key studies in which favorable sexual-function and testosterone-axis findings appeared together (40). By contrast, Steels et al. a fenugreek-mineral coformulation study—showed favorable sexual-function outcomes without a clear increase in serum testosterone, suggesting that symptom improvement and testosterone change are not necessarily synchronous (45). Hausenblas et al. reported directional improvements in AMS, grip strength, and health-related quality of life, but did not provide blood testosterone or estradiol data and can therefore only be treated as symptom- or function-related evidence (48). The mixed-extract study of Park et al. and the endurance-performance study of Thakurdesai et al. further illustrate that broader male-health evidence may help define the possible scope of fenugreek-related effects, but should not alter the evidential weight of the primary testosterone-axis conclusion (47, 49).

### 3.8 Safety outcomes

Safety outcomes were reported inconsistently across the included evidence. Eight studies provided explicit safety-related information, but the scope varied from general tolerability statements to selected biomarkers such as PSA, prolactin, liver function, renal function, or other routine laboratory measures. Reported non-serious adverse events were uncommon and not consistently categorized; several articles simply stated that no adverse events or no serious adverse events occurred. No included article reported a serious adverse event attributable to fenugreek, but the short follow-up, small sample sizes, and inconsistent adverse-event reporting prevent firm conclusions about long-term safety (Supplementary Table S3).

Supplementary material provides the safety-reporting summary, full quantitative results, deletion and leave-one-out sensitivity analyses, dose/duration and subgroup feasibility maps, and the reporting-basis and data-handling rationale (Supplementary Tables S3–S7). Dose–response analysis, meta-regression, formal subgroup-effect testing, funnel plots, and trial sequential analysis were not included in the main manuscript; their boundaries and rationale are described in the Methods and Supplementary Tables S6, S7. This presentation strategy was intended to avoid misinterpreting exploratory or boundary findings as core evidence equivalent to total testosterone or free testosterone. Because fewer than 10 studies contributed to each primary pooled outcome, Begg’s test, Egger’s test, funnel-plot asymmetry assessment, and trim-and-fill adjustment were not performed. This does not mean that publication bias was ignored; rather, formally precise but practically unreliable inferences were avoided in a sparse evidence setting. The potential risk of publication bias was reflected in the GRADE assessment as “suspected publication bias” and was interpreted in conjunction with industry sponsorship, small-study effects, and the proprietary-product research environment.

## 4 Discussion

This systematic review indicates that oral fenugreek-derived preparations are associated with a small increase in total testosterone in adult men, whereas the pooled estimate for free testosterone is imprecise and heterogeneous. Because the certainty of evidence for both primary testosterone-axis outcomes was very low, these findings should be interpreted cautiously as limited biochemical signals rather than as evidence that fenugreek can reliably increase clinically meaningful androgen exposure. Favorable directions were also observed for some symptom, sexual-function, body-composition, and performance outcomes, but these outcomes cannot substitute for the primary testosterone-axis evidence and do not establish that their effects are mediated by testosterone change.

The pattern of a small favorable total-testosterone signal alongside unstable free-testosterone results may arise from at least three sources. First, the included studies differed substantially in how free testosterone was measured or estimated: some used calculated free testosterone, some reported the free testosterone index (FTI), and others reported salivary testosterone or did not report SHBG (40, 44, 46). Calculated free testosterone may be practically useful under certain circumstances, but it is not interchangeable with every form of “free testosterone” reported across studies (13, 50–53). Second, study-level outcome patterns were not concordant across exercise, BPH, and healthy aging settings. Third, the biological relationship between total testosterone and free testosterone is not linear and is influenced by SHBG concentrations, sampling time, and baseline population status (12, 14). A small increase in total testosterone therefore does not automatically imply a stable benefit for free testosterone.

The magnitude of the pooled effect also warrants careful interpretation. The pooled effect size for total testosterone was SMD = 0.25, representing a small effect. Because different assay methods and units were used across studies, SMD was an appropriate statistical choice, but a standardized effect does not correspond to an absolute change in serum testosterone in a fixed unit. In routine practice, this small and very-low-certainty standardized signal is unlikely to justify a claim of clinically meaningful testosterone improvement. SMDs are influenced by the underlying distribution, within-study variability, and between-study heterogeneity (54). The result is therefore better described as a small favorable standardized signal rather than as evidence of a testosterone increase reaching a particular clinical threshold. The free-testosterone estimate, which crossed the null, further limits inference about androgen bioavailability.

From a biological and formulation perspective, fenugreek contains multiple constituents potentially relevant to metabolism, inflammation, endocrine function, or exercise performance, including steroidal sapogenins such as diosgenin, saponins, trigonelline, 4-hydroxyisoleucine, flavonoids, and related compounds (1, 2, 55, 56). Pharmacological, extraction, quality-control, functional-food, performance, and testosterone-booster reviews often frame fenugreek in terms of potentially active ingredients (6, 11, 57–61). However, mechanistic plausibility is not equivalent to a stable and generalizable testosterone effect in randomized human trials. The included studies involved different proprietary extracts, doses, standardization strategies, training backgrounds, and health states, and the results for total testosterone and free testosterone were not concordant.

Several mechanisms have been proposed, but current evidence remains indirect. Steroidal saponins and sapogenins such as diosgenin are often hypothesized to influence steroid metabolism, androgen signaling, or enzyme activity; trigonelline and 4-hydroxyisoleucine may affect insulin sensitivity, energy metabolism, and metabolic stress; antioxidant and anti-inflammatory activity may modify the endocrine milieu; and SHBG modulation could alter the relationship between total and free testosterone (1–6, 55–61). However, the included randomized trials were not designed to test these pathways, and the nonconcordant total- and free-testosterone findings argue against a simple direct androgen-boosting mechanism.

The evidence also does not justify treating “fenugreek” as a uniform intervention entity. Different trials evaluated Testofen, Furosap, AlphaFen, FEDE, IND9, a fenugreek-mineral coformulation, and a mixed TFGL/*Lespedeza cuneata* extract, and the standardized components and formulation backgrounds were not the same (40, 41, 45–49). Differences in extraction methods, standardization procedures, delivery systems, and concentrations of steroidal saponins, diosgenin, protodioscin, trigonelline, 4-hydroxyisoleucine, and glycosides may therefore contribute to between-study heterogeneity. Recent work on green-seeded fenugreek genotypes also illustrates that genotype and seed characteristics can affect 4-hydroxyisoleucine, diosgenin, chlorophyll, and saponin content (4, 5). These products represented proprietary saponin-rich extracts, protodioscin-enriched extracts, liposomal delivery systems, standardized combinations containing 4-hydroxyisoleucine, trigonelline, and glycosides, an acute single-dose crossover product, mineral coformulations, or mixed botanical extracts. Some studies also involved exercise-training cointerventions or multidose matrices. Even when these products shared a fenugreek source, their chemical composition, bioavailability, cointervention context, and target populations could still differ. Formulation differences were therefore treated here as boundaries of indirectness, applicability, and mechanistic interpretation rather than compressed into a single uniform “fenugreek effect.”

Diosgenin also requires a balanced reproductive-safety interpretation. Preclinical literature has reported potentially protective effects on testicular oxidative stress, inflammation, steroidogenesis, or spermatogenic injury, whereas other experimental studies have raised concerns that high-dose diosgenin or certain fenugreek-derived exposures may adversely affect reproductive hormones, spermatogenesis, sperm quality, or fertility-related parameters (20, 55, 62, 63). These findings are model-, dose-, and formulation-dependent and should not be directly extrapolated to short-term human supplementation trials. Importantly, a change in circulating testosterone does not necessarily imply improved semen quality, fertility, or other clinically meaningful reproductive endpoints.

Regarding applicability, the current evidence does not directly address whether fenugreek is appropriate for men with confirmed low testosterone or clinical hypogonadism. Most trials were conducted in healthy participants, men in exercise-training settings, athletes, recreationally active men, middle-aged or older men, men with BPH, or symptom-oriented populations, and baseline androgen status, symptom burden, and assay methods were not standardized. Endocrine Society guidance emphasizes that diagnosis and treatment decisions for male hypogonadism should be based on symptoms, signs, and clearly and consistently low testosterone concentrations rather than on a single measurement or supplement response (16). The current findings are therefore more useful for evidence appraisal and future study design than for direct treatment decisions, and should not be translated into commercial language implying that fenugreek “reliably boosts testosterone.”

Risk of bias and certainty of evidence further constrain the strength of inference. Most studies were judged as having some concerns, two were at high risk of bias, and none were at low risk overall. Selection of the reported result, limited protocol or registry traceability, multiple outcomes and time points, missing outcome data, and incompletely reported hormone-analysis denominators all reduce confidence in the pooled estimates. The GRADE assessment rated both total testosterone and free testosterone as very low certainty. Because fewer than 10 studies contributed to each primary synthesis, funnel plots and Egger’s test were not performed, consistent with the principle that funnel-plot asymmetry is difficult to interpret in sparse evidence settings (64–66).

Strengths of this review include broad database and registry searches, prespecified evidence layering, outcome-level synthesis, sensitivity analyses, and use of RoB 2 and GRADE. Important limitations remain. First, the number of included studies was small, with only five to six studies contributing to each primary pooled outcome. Second, the marked heterogeneity in populations, proprietary formulations, extraction and standardization procedures, and outcomes limits extrapolation to men with confirmed testosterone deficiency or other clinical populations. Third, some outcomes depended on unit conversion, SEM-to-SD conversion, calculated free testosterone, salivary measures, or reporting-basis judgments, all of which add uncertainty to data handling. Fourth, safety reporting was incomplete, adverse-event denominators and definitions were often unclear, most studies were small and short in duration, and long-term risks cannot be excluded (62, 63). Fifth, industry sponsorship or company involvement was common, which increases concern about selective publication or selective outcome reporting. Finally, the evidence relied mainly on surrogate hormonal outcomes rather than clinically meaningful reproductive endpoints such as semen quality or fertility; related and supplementary outcomes provide a broader male-health context but cannot replace the primary testosterone-axis evidence.

The current evidence does not support claims that fenugreek can reliably or clinically meaningfully increase testosterone (7–9). For users and clinicians, the most appropriate summary is that randomized trials suggest a small favorable direction for total testosterone, but the evidence is very uncertain and free testosterone does not show a stable benefit. Future trials should register protocols before recruitment; use standardized and chemically characterized fenugreek preparations; harmonize morning testosterone assay methods; measure SHBG and free testosterone using standardized protocols; transparently report sample-size handling and all prespecified outcomes; recruit adequately powered multicenter samples, including clinically hypogonadal populations where appropriate; extend intervention and follow-up durations; and give comprehensive safety endpoints, including prostate-specific antigen, liver function, renal function, adverse events, and reproductive endpoints, the same priority as hormone outcomes.

## 5 Conclusion

Oral fenugreek-derived preparations may be associated with a small increase in total testosterone in adult men, but the certainty of evidence is very low and no stable free-testosterone benefit was demonstrated. Symptom, sexual-function, body-composition, and performance outcomes may provide related contextual information, but they cannot substitute for the primary testosterone-axis evidence. Current evidence should be interpreted as surrogate biochemical evidence rather than proof of improved androgen bioavailability, fertility, or long-term safety, and it does not support strong clinical or commercial testosterone-boosting claims. Future randomized trials should prioritize prospective registration, chemically characterized formulation standardization, harmonized testosterone and SHBG/free-testosterone assays, clinically relevant populations, longer follow-up, and prespecified safety and reproductive endpoints.

## Data Availability

The original contributions presented in the study are included in the article/Supplementary material, further inquiries can be directed to the corresponding authors.
