# Supplementary material for: Effects of oral fenugreek-derived preparations on testosterone-related outcomes in adult men: a systematic review and meta-analysis of randomized placebo-controlled trials
Source: Front Nutr. 2026 Aug 19;13:1903508. doi: 10.3389/fnut.2026.1903508 (PMC13534118; doi:10.3389/fnut.2026.1903508)
Supplement: Supplementary file 1 [file Table_1.DOCX]

Supplementary Material

# Supplementary Material

This supplementary file provides the exact search strategies, registry/watchlist disposition record, complete quantitative results, sensitivity analyses, and prespecified exploratory evidence maps supporting the systematic review and meta-analysis.

## Supplementary Figures

No supplementary figures are included.

## Supplementary Tables

### Table S1. Exact search strategies and record counts.

All bibliographic and registry sources were last refreshed on 2026-05-11.

| **Source** | **Search strategy** |
| --- | --- |
| PubMed main formal RCT search (170 records) | (fenugreek[tiab] OR "Trigonella foenum-graecum"[tiab] OR "Trigonella foenum graecum"[tiab] OR Testofen[tiab] OR Furosap[tiab] OR "Fenu-FG"[tiab] OR FenuFG[tiab] OR Trigozim[tiab] OR Testosurge[tiab]) AND (randomized controlled trial[pt] OR controlled clinical trial[pt] OR clinical trial[pt] OR randomized[tiab] OR randomised[tiab] OR placebo[tiab] OR crossover[tiab] OR "cross-over"[tiab] OR "double blind"[tiab] OR "double-blind"[tiab]) |
| PubMed testosterone-axis focus (62 records) | (fenugreek[tiab] OR "Trigonella foenum-graecum"[tiab] OR "Trigonella foenum graecum"[tiab] OR Testofen[tiab] OR Furosap[tiab] OR "Fenu-FG"[tiab] OR FenuFG[tiab] OR Trigozim[tiab] OR Testosurge[tiab]) AND (testosterone[tiab] OR androgen[tiab] OR libido[tiab] OR sexual[tiab] OR erectile[tiab] OR prostate[tiab] OR BPH[tiab] OR PSA[tiab] OR SHBG[tiab] OR "body composition"[tiab] OR strength[tiab] OR resistance[tiab] OR exercise[tiab] OR muscle[tiab] OR performance[tiab]) AND (randomized controlled trial[pt] OR controlled clinical trial[pt] OR clinical trial[pt] OR randomized[tiab] OR randomised[tiab] OR placebo[tiab] OR crossover[tiab] OR "cross-over"[tiab] OR "double blind"[tiab] OR "double-blind"[tiab]) |
| PubMed synthesis competitor check (255 records) | (fenugreek[tiab] OR "Trigonella foenum-graecum"[tiab] OR "Trigonella foenum graecum"[tiab] OR Testofen[tiab] OR Furosap[tiab] OR "Fenu-FG"[tiab] OR FenuFG[tiab] OR Trigozim[tiab] OR Testosurge[tiab]) AND (meta-analysis[pt] OR systematic review[pt] OR review[pt] OR meta-analysis[tiab] OR systematic review[tiab]) |
| PubMed recent synthesis competitor check (25 records) | (fenugreek[tiab] OR "Trigonella foenum-graecum"[tiab] OR "Trigonella foenum graecum"[tiab] OR Testofen[tiab] OR Furosap[tiab] OR "Fenu-FG"[tiab] OR FenuFG[tiab] OR Trigozim[tiab] OR Testosurge[tiab]) AND (testosterone[tiab] OR androgen[tiab] OR libido[tiab] OR sexual[tiab] OR erectile[tiab] OR prostate[tiab] OR BPH[tiab] OR PSA[tiab] OR SHBG[tiab] OR "body composition"[tiab] OR strength[tiab] OR resistance[tiab] OR exercise[tiab] OR muscle[tiab] OR performance[tiab]) AND (meta-analysis[pt] OR systematic review[pt] OR review[pt] OR meta-analysis[tiab] OR systematic review[tiab]) AND ("2023"[dp] : "2026"[dp]) |
| Embase main (460 records) | ('fenugreek':ti,ab,kw OR trigonella:ti,ab,kw OR 'trigonella foenum-graecum':ti,ab,kw OR 'trigonella foenum graecum':ti,ab,kw OR 'fenugreek extract':ti,ab,kw OR 'fenugreek seed extract':ti,ab,kw OR 'standardized fenugreek extract':ti,ab,kw OR 'fenugreek glycoside fraction':ti,ab,kw OR testofen:ti,ab,kw OR furosap:ti,ab,kw OR 'fenu-fg':ti,ab,kw OR fenufg:ti,ab,kw OR trigozim:ti,ab,kw OR testosurge:ti,ab,kw OR fenuside:ti,ab,kw OR fenusmart:ti,ab,kw OR alphafen:ti,ab,kw OR indus810:ti,ab,kw OR libifem:ti,ab,kw) AND (random*:ti,ab,kw OR placebo:ti,ab,kw OR crossover:ti,ab,kw OR 'cross-over':ti,ab,kw OR 'double blind':ti,ab,kw OR 'double-blind':ti,ab,kw OR 'clinical trial':ti,ab,kw OR 'controlled clinical trial':ti,ab,kw OR 'randomized controlled trial':ti,ab,kw) |
| Embase author rescue (68 records) | ('fenugreek':ti,ab,kw OR trigonella:ti,ab,kw OR 'trigonella foenum-graecum':ti,ab,kw OR 'fenugreek extract':ti,ab,kw OR 'fenugreek seed extract':ti,ab,kw OR testofen:ti,ab,kw OR furosap:ti,ab,kw OR 'fenu-fg':ti,ab,kw OR fenufg:ti,ab,kw OR trigozim:ti,ab,kw OR testosurge:ti,ab,kw OR fenuside:ti,ab,kw OR fenusmart:ti,ab,kw OR alphafen:ti,ab,kw OR indus810:ti,ab,kw OR libifem:ti,ab,kw) AND (wankhede:au OR poole:au OR rao:au OR wilborn:au OR steels:au OR hausenblas:au OR guo:au OR 'lee-odegard':au) |
| Cochrane CENTRAL main, Trials tab only (375 records) | fenugreek OR trigonella OR "Trigonella foenum-graecum" OR "Trigonella foenum graecum" OR "fenugreek extract" OR "fenugreek seed extract" OR "standardized fenugreek extract" OR "fenugreek glycoside fraction" OR Testofen OR Furosap OR "Fenu-FG" OR FenuFG OR Trigozim OR Testosurge OR Fenuside OR FenuSMART OR AlphaFen OR INDUS810 OR Libifem |
| Cochrane CENTRAL author rescue, Trials tab only (24 records) | #1 Title Abstract Keyword: fenugreek OR trigonella OR "Trigonella foenum-graecum" OR "Trigonella foenum graecum" OR "fenugreek extract" OR "fenugreek seed extract" OR "standardized fenugreek extract" OR "fenugreek glycoside fraction" OR Testofen OR Furosap OR "Fenu-FG" OR FenuFG OR Trigozim OR Testosurge OR Fenuside OR FenuSMART OR AlphaFen OR INDUS810 OR Libifem; #2 Author: Wankhede OR Poole OR Rao OR Wilborn OR Steels OR Hausenblas OR Guo OR "Lee-Odegard"; #3 = #1 AND #2 |
| Web of Science main (394 records) | TS=(fenugreek OR trigonella OR "Trigonella foenum-graecum" OR "Trigonella foenum graecum" OR "fenugreek extract" OR "fenugreek seed extract" OR "standardized fenugreek extract" OR "fenugreek glycoside fraction" OR Testofen OR Furosap OR "Fenu-FG" OR FenuFG OR Trigozim OR Testosurge OR Fenuside OR FenuSMART OR AlphaFen OR INDUS810 OR Libifem) AND TS=(random* OR placebo OR crossover OR "cross-over" OR "double blind" OR "double-blind" OR "clinical trial" OR "controlled clinical trial" OR "randomized controlled trial") |
| Web of Science author rescue (82 records) | TS=(fenugreek OR trigonella OR "Trigonella foenum-graecum" OR "fenugreek extract" OR "fenugreek seed extract" OR Testofen OR Furosap OR "Fenu-FG") AND AU=(Wankhede OR Poole OR Rao OR Wilborn OR Steels OR Hausenblas OR Guo OR "Lee-Odegard") |
| Scopus main (946 records) | TITLE-ABS-KEY(fenugreek OR trigonella OR "Trigonella foenum-graecum" OR "Trigonella foenum graecum" OR "fenugreek extract" OR "fenugreek seed extract" OR "standardized fenugreek extract" OR "fenugreek glycoside fraction" OR Testofen OR Furosap OR "Fenu-FG" OR FenuFG OR Trigozim OR Testosurge OR Fenuside OR FenuSMART OR AlphaFen OR INDUS810 OR Libifem) AND TITLE-ABS-KEY(random* OR placebo OR crossover OR "cross-over" OR "double blind" OR "double-blind" OR "clinical trial" OR "controlled clinical trial" OR "randomized controlled trial") |
| Scopus author rescue (127 records) | TITLE-ABS-KEY(fenugreek OR trigonella OR "Trigonella foenum-graecum" OR "fenugreek extract" OR "fenugreek seed extract" OR Testofen OR Furosap OR "Fenu-FG") AND (AUTHOR-NAME(wankhede) OR AUTHOR-NAME(poole) OR AUTHOR-NAME(rao) OR AUTHOR-NAME(wilborn) OR AUTHOR-NAME(steels) OR AUTHOR-NAME(hausenblas) OR AUTHOR-NAME(guo) OR AUTHOR-NAME(odegard)) |
| ClinicalTrials.gov API v2 refresh (60 records) | fenugreek OR Testofen OR Furosap OR Trigonella |
| ClinicalTrials.gov manual RIS export (33 records) | fenugreek OR Testofen OR Furosap OR Trigonella |
| WHO ICTRP main (26 records) | fenugreek OR trigonella OR "Trigonella foenum-graecum" OR Testofen OR Furosap OR "Fenu-FG" OR FenuFG OR Trigozim OR Testosurge OR Fenuside OR FenuSMART OR AlphaFen OR INDUS810 OR Libifem |
| WHO ICTRP product rescue (10 records) | Testofen OR Furosap OR "Fenu-FG" OR FenuFG OR Trigozim OR Testosurge OR Fenuside OR FenuSMART OR AlphaFen OR INDUS810 OR Libifem |

Search-results summary used for the current record-level PRISMA workflow:

| **Source** | **Records identified** |
| --- | --- |
| PubMed broad RCT search | 170 |
| PubMed testosterone-axis focus | 62 |
| PubMed synthesis competitor check | 255 |
| PubMed recent synthesis competitor check | 25 |
| Embase main | 460 |
| Embase author rescue | 68 |
| CENTRAL main, Trials tab only | 375 |
| CENTRAL author rescue, Trials tab only | 24 |
| Web of Science main | 394 |
| Web of Science author rescue | 82 |
| Scopus main | 946 |
| Scopus author rescue | 127 |
| ClinicalTrials.gov API v2 refresh | 60 |
| ClinicalTrials.gov manual RIS export | 33 |
| WHO ICTRP main | 26 |
| WHO ICTRP product rescue | 10 |
| Total source-record rows before preliminary deduplication | 2984 |

- records removed by preliminary DOI / PMID / registry ID / title dedupe: 1403
- title/abstract records screened after preliminary deduplication: 1581
- title/abstract records excluded: 1549
- reports sought for retrieval / eligibility check: 14
- reports excluded after full-text/report review: 1
- reports/study units included after P8: 13
- registry/watchlist records retained outside the published-study denominator: 18

Notes:

- CENTRAL exports were restricted to the Trials tab, and Cochrane Reviews were not imported into the trial pool.
- ClinicalTrials.gov API v2 remained the primary ClinicalTrials.gov registry source; the manually downloaded RIS was retained as a supplementary registry export.
- The registry/watchlist records remained outside the denominator of published randomized trials and outside the quantitative synthesis denominator.

### Table S2. Registry watchlist records retained outside the published-study denominator.

The only full-text/report-level exclusion after eligibility review was the duplicate or secondary Guo 2018 Furosap abstract report (D00415). The 18 rows below were registry or registry-linked watchlist records retained outside the denominator of published randomized trials and outside the quantitative synthesis denominator.

| **Registry/watchlist records** | **Reason retained outside the published-study denominator** |
| --- | --- |
| Evaluating the Efficacy and Safety of Testosurge Capsules for Hormonal Balance in Healthy Adult Males: a Double-Blind, Placebo-Controlled Study (1) | Testosurge male testosterone registry; fenugreek anchor not visible in title/abstract. |
| Clinical trial on lower urinary tract symptoms and male menopausal symptoms (2) | 2024 CTRI BPH/male-menopause fenugreek placebo registry. |
| Effect of Testofen on Erectile Function in an Adult Male Population (3) | NCT05232279 Testofen ED trial; publication not in current source pool. |
| Effects of INDUS810 on Body Composition, Muscular Performance, and Training Adaptations During 12-weeks of Supplementation and Concurrent Training (4) | Embase ClinicalTrials.gov record linked to NCT03850106. |
| Effects of INDUS810 on Body Composition, Muscular Performance, and Training Adaptations (5) | NCT03850106 INDUS810 registry; no posted publication in current pool. |
| Effect of food intake containing fenugreek on testosterone (6) | JPRN fenugreek/testosterone registry. |
| The effects of 8 weeks of a Fenugreek extract supplementation in combination with exercise training on exercise capacity in healthy young males (7) | ACTRN exercise-capacity registry; likely linked to 2024 FEDE endurance article. |
| The Efficacy of Fenugreek Supplementation on Men’s Health (8) | AlphaFen men’s-health registry; linked to Hausenblas 2020 signal. |
| Oral Testofen, L-citrulline, Resveratrol, and Caffeine supplementation study for sexual function: randomized, double blind, placebo-controlled crossover pilot study. (9) | Testofen/L-citrulline/resveratrol/caffeine crossover registry; nonseparable combo. |
| Efficacy of Fenugreek Seed and Lespedeza Cuneata in TDS (10) | TFG/Lespedeza TDS registry; linked to Park 2018 publication. |
| Efficacy and Safety of Fenugreek Extract on Markers of Muscle Damage and Inflammation in Untrained Males (11) | NCT03318731 muscle-damage registry; noncore outcome boundary. |
| Evaluation of Furosap in Human Volunteers (12) | Furosap volunteer registry; likely open-label context, not hard-threshold evidence. |
| A double-blind, randomised, placebo-controlled interventional study to evaluate the effect of orally-dosed herbal extract, Testofen (Trigonella foenum-graecum) on muscle strength, endurance and body composition in men aged between 25 and 45 years. (13) | Testofen muscle-strength/body-composition registry; likely linked to calisthenics/performance evidence. |
| The Efficacy Study of FUROSAP: A Testosterone Booster Supplement In Humans. (14) | Furosap testosterone-booster registry; no placebo-controlled evidence visible. |
| A double-blind, randomised, placebo-controlled study to evaluate the effect of an orally-dosed herbal extract, Testofen, for the treatment of Benign Prostatic Hyperplasia (BPH) symptoms in otherwise healthy males. (15) | BPH Testofen registry; linked to Rao 2020 BPH publication. |
| A double-blind, randomised, placebo-controlled study to evaluate the effect of an orally-dosed herbal formulation containing Testofen, on symptoms of Andropause and serum testosterone levels in otherwise healthy males aged between 45-75 years. (16) | Andropause/Testofen registry; linked to Rao 2016 publication. |
| A double-blind, randomised, placebo-controlled study to evaluate the effect of an orally-dosed herbal formulation containing Testofen, on sexual function, performance, and serum testosterone levels in healthy males. (17) | Testofen/mineral sexual-function registry; linked to Steels 2011. |
| Clinical study on TESTODAAT in male subjects during resistance training (18) | TESTODAAT male resistance-training registry. |

**References**

1. CTRI/2024/10/075195. Evaluating the Efficacy and Safety of Testosurge Capsules for Hormonal Balance in Healthy Adult Males: a Double-Blind, Placebo-Controlled Study. WHO ICTRP trialsearch entry; CENTRAL accession CN-02775440.
2. Daehan chemtech co., Ltd. Clinical trial on lower urinary tract symptoms and male menopausal symptoms. CTRI. CTRI/2024/05/067789.
3. RDC Clinical Pty Ltd. Effect of Testofen on Erectile Function in an Adult Male Population. ClinicalTrials.gov API v2. NCT05232279. Linked publication status at the current search update: none identified in the screened source pool as of 2026-05-11.
4. Effects of INDUS810 on Body Composition, Muscular Performance, and Training Adaptations During 12-weeks of Supplementation and Concurrent Training. Embase-imported clinicaltrials.gov registry record linked to NCT03850106. This watchlist row was the Embase-imported clinicaltrials.gov duplicate linked to NCT03850106 and was retained to document the registry-level duplicate chain.
5. The Center for Applied Health Sciences, LLC. Effects of INDUS810 on Body Composition, Muscular Performance, and Training Adaptations. ClinicalTrials.gov API v2. NCT03850106. Registry status at the current search update: completed registry record without a posted results paper in the screened source pool.
6. Japan clinical trial assosiation. Effect of food intake containing fenugreek on testosterone. JPRN. JPRN-UMIN000038786.
7. ACTRN12618001356257. The effects of 8 weeks of a Fenugreek extract supplementation in combination with exercise training on exercise capacity in healthy young males. WHO ICTRP trialsearch entry; CENTRAL accession CN-02445356.
8. Jacksonville University. The Efficacy of Fenugreek Supplementation on Men’s Health. ClinicalTrials.gov API v2. NCT03528538.
9. Juntendo University Urayasu Hospital. Oral Testofen, L-citrulline, Resveratrol, and Caffeine supplementation study for sexual function: randomized, double blind, placebo-controlled crossover pilot study. JPRN. JPRN-UMIN000028816.
10. Pusan National University Hospital. Efficacy of Fenugreek Seed and Lespedeza Cuneata in TDS. ClinicalTrials.gov API v2. NCT03057899.
11. University of Mary Hardin-Baylor. Efficacy and Safety of Fenugreek Extract on Markers of Muscle Damage and Inflammation in Untrained Males. ClinicalTrials.gov API v2. NCT03318731.
12. Chemical Resources. Evaluation of Furosap in Human Volunteers. ClinicalTrials.gov API v2. NCT02748005.
13. Integrated Health Global Pty Ltd. A double-blind, randomised, placebo-controlled interventional study to evaluate the effect of orally-dosed herbal extract, Testofen (Trigonella foenum-graecum) on muscle strength, endurance and body composition in men aged between 25 and 45 years. ANZCTR. ACTRN12616000938404.
14. Chemical Resources. The Efficacy Study of FUROSAP: A Testosterone Booster Supplement In Humans. ClinicalTrials.gov. NCT02702882.
15. Amanda Rao. A double-blind, randomised, placebo-controlled study to evaluate the effect of an orally-dosed herbal extract, Testofen, for the treatment of Benign Prostatic Hyperplasia (BPH) symptoms in otherwise healthy males. ANZCTR. ACTRN12615001242516.
16. AZPA International. A double-blind, randomised, placebo-controlled study to evaluate the effect of an orally-dosed herbal formulation containing Testofen, on symptoms of Andropause and serum testosterone levels in otherwise healthy males aged between 45-75 years. ANZCTR. ACTRN12613000925741.
17. AZPA International. A double-blind, randomised, placebo-controlled study to evaluate the effect of an orally-dosed herbal formulation containing Testofen, on sexual function, performance, and serum testosterone levels in healthy males. ANZCTR. ACTRN12611000830998.
18. Clinical study on TESTODAAT in male subjects during resistance training. CTRI/2021/07/034548.

**Table S3. Safety and adverse-event reporting across included studies.**

This table summarizes whether and how each included study reported safety information. It is a reporting summary rather than a pooled estimate of safety risk. AE, adverse event; PSA, prostate-specific antigen; SHBG, sex hormone-binding globulin.

| **Study unit** | **Safety data reported** | **Brief safety summary** |
| --- | --- | --- |
| Wilborn 2010 | No structured AE or laboratory safety summary identified. | Safety inference limited by absent structured reporting. |
| Poole 2010 | No structured AE or laboratory safety summary identified. | Safety inference limited by absent structured reporting. |
| Wankhede 2016 | General tolerability/safety statement. | Reported as safe and well tolerated; detailed AE types were not tabulated. |
| Rao 2016 Testofen | Hormone-related biomarkers, including SHBG and prolactin; limited AE detail. | No structured AE summary available for extraction. |
| Guo 2018 Furosap | No structured AE or laboratory safety summary identified. | Safety inference limited by absent structured reporting. |
| Rao-Mallard-Grant 2020 | Limited safety information in the extracted report. | No structured AE summary available for extraction. |
| Rao 2020 BPH | PSA and prostate-function-related safety outcomes. | No serious safety signal described; applicability is BPH-specific. |
| Lee-Odegard 2024 | Safety/tolerability information in a multidose trial. | No serious safety concern described; non-serious AE detail was limited. |
| Steels 2011 | Safety-related biomarkers including prolactin and PSA. | No serious safety concern described; fenugreek-mineral coformulation. |
| Mokashi 2014 IND9 | Acute tolerability after single-dose exposure. | No acute safety concern described; not informative for long-term safety. |
| Park 2018 TFGL | Safety outcomes for mixed Trigonella/Lespedeza extract. | No serious safety concern described; fenugreek-specific safety cannot be isolated. |
| Hausenblas 2020 | Adverse-event statement. | No adverse events reported in the article. |
| Thakurdesai 2024 FEDE | Routine safety laboratory outcomes and tolerability. | No serious safety concern described; no testosterone-axis outcome reported. |

### Table S4. Complete quantitative results summary.

Outcome-level quantitative results retained for the full supplementary presentation. Values are effect estimates with 95% CIs.

| **Outcome** | **Role** | **Metric** | **Studies, k** | **Effect estimate (95% CI)** | **I² (%)** | **Note** |
| --- | --- | --- | --- | --- | --- | --- |
| Total testosterone | Main | Hedges g | 6 | 0.25 (0.02 to 0.48) | 0.0 | Main testosterone-axis pooled outcome |
| Free testosterone | Main | Hedges g | 5 | 0.08 (-0.48 to 0.63) | 66.7 | Main testosterone-axis pooled outcome |
| AMS psychology | Secondary | MD | 2 | -1.31 (-2.37 to -0.25) | 0.0 | Symptom-domain outcome |
| AMS sexual | Secondary | MD | 2 | -0.95 (-2.45 to 0.55) | 80.6 | Symptom-domain outcome |
| AMS somatic | Secondary | MD | 2 | -2.22 (-3.52 to -0.91) | 0.0 | Symptom-domain outcome |
| AMS total | Secondary | MD | 2 | -4.56 (-7.98 to -1.15) | 26.5 | Symptom-domain outcome |
| DISF total | Secondary | GIV MD | 2 | 16.97 (6.06 to 27.88) | 74.9 | Sexual-function outcome with coformulation boundary |
| SHBG | Supplementary | MD (nmol/L) | 3 | 0.36 (-4.71 to 5.44) | 9.7 | Supplementary hormone-related outcome |
| Fat-free mass | Supplementary | MD (kg) | 2 | 1.03 (-3.25 to 5.31) | 0.0 | Supplementary body-composition outcome |
| Body-fat percentage | Supplementary | MD (percentage points) | 4 | -1.37 (-2.13 to -0.61) | 27.3 | Supplementary body-composition outcome |
| Bench-press 1RM | Supplementary | MD (kg) | 4 | 4.86 (1.71 to 8.02) | 0.0 | Supplementary performance outcome |
| Leg-press 1RM | Supplementary | MD (kg) | 4 | 15.64 (-6.11 to 37.38) | 85.5 | Supplementary performance outcome |
| Prolactin | Supplementary | MD (ug/L) | 2 | -0.30 (-1.47 to 0.88) | 50.2 | Supplementary hormone-related outcome with source-label caveat |

### Table S5. Sensitivity and leave-one-out analyses.

Prespecified deletion sensitivity analyses and leave-one-out diagnostics for the main testosterone-axis outcomes and selected supplementary outcomes.

| **Outcome and analysis** | **Analysis type** | **Studies, k** | **Effect estimate (95% CI)** | **I² (%)** | **Interpretation note** |
| --- | --- | --- | --- | --- | --- |
| Total testosterone: exclude benign prostatic hyperplasia population | Deletion sensitivity | 5 | 0.34 (0.17 to 0.52) | 0.0 | Main testosterone-axis sensitivity analysis |
| Total testosterone: exclude exercise or athlete setting | Deletion sensitivity | 2 | 0.11 (-1.98 to 2.21) | 18.1 | Main testosterone-axis sensitivity analysis |
| Total testosterone: exclude product or formulation boundary | Deletion sensitivity | 5 | 0.23 (-0.03 to 0.49) | 0.0 | Main testosterone-axis sensitivity analysis |
| Total testosterone: exclude Rao calisthenics multiarm dose boundary | Deletion sensitivity | 5 | 0.21 (-0.04 to 0.46) | 0.0 | Main testosterone-axis sensitivity analysis |
| Total testosterone: exclude SEM-to-SD rows | Deletion sensitivity | 5 | 0.26 (-0.05 to 0.56) | 2.1 | Main testosterone-axis sensitivity analysis |
| Total testosterone: leave-one-out | Leave-one-out | 6 | 0.25 (0.02 to 0.48) | 0.0 | Main testosterone-axis diagnostic |
| Free testosterone: exclude benign prostatic hyperplasia population | Deletion sensitivity | 4 | 0.05 (-0.79 to 0.89) | 74.7 | Main testosterone-axis sensitivity analysis |
| Free testosterone: exclude exercise or athlete setting | Deletion sensitivity | 2 | 0.29 (-1.61 to 2.19) | 0.0 | Main testosterone-axis sensitivity analysis |
| Free testosterone: exclude product or formulation boundary | Deletion sensitivity | 4 | 0.27 (0.04 to 0.49) | 0.0 | Main testosterone-axis sensitivity analysis |
| Free testosterone: exclude Rao calisthenics multiarm dose boundary | Deletion sensitivity | 4 | 0.03 (-0.78 to 0.84) | 75.7 | Main testosterone-axis sensitivity analysis |
| Free testosterone: leave-one-out | Leave-one-out | 5 | 0.08 (-0.48 to 0.63) | 66.7 | Main testosterone-axis diagnostic |
| SHBG: leave-one-out | Leave-one-out | 3 | 0.36 (-4.71 to 5.44) | 9.7 | Supplementary diagnostic |
| Body-fat percentage: leave-one-out | Leave-one-out | 4 | -1.37 (-2.13 to -0.61) | 27.3 | Supplementary diagnostic |
| Bench-press 1RM: leave-one-out | Leave-one-out | 4 | 4.86 (1.71 to 8.02) | 0.0 | Supplementary diagnostic |
| Leg-press 1RM: leave-one-out | Leave-one-out | 4 | 15.64 (-6.11 to 37.38) | 85.5 | Supplementary diagnostic |

### Table S6. Exploratory dose-duration and subgroup evidence map.

These evidence maps describe why formal dose-response, meta-regression, and subgroup-effect models were not performed. They should be interpreted as transparency tables rather than confirmatory effect-modification analyses.

#### Table S6A. Dose-duration evidence map.

| **Evidence question** | **Outcomes considered** | **Evidence available** | **Presentation** | **Rationale** |
| --- | --- | --- | --- | --- |
| Dose-duration pattern for total testosterone | Total testosterone | Six main effect rows, mainly around 500 to 600 mg/day, with additional wider dose context from a nonselected 300 mg Rao 2020 arm and a combined-dose Lee-Odegard model output | Descriptive evidence map only | The dose range was narrow, formulations and populations differed, Rao 2020 shared a placebo group across dose arms, and a continuous slope would be over-interpreted |
| Dose-duration pattern for free testosterone | Free testosterone | Five main effect rows, with wider context from Rao 2020 and the acute IND9 crossover study | Descriptive evidence map only | Unit/source trace issues, shared-placebo dose arms, and acute crossover evidence limit comparability with chronic parallel-group RCTs |
| Dose-duration pattern for SHBG | SHBG | Three unit-aligned rows, with Rao 2020 dose context | Descriptive evidence map only | The number of studies was too small for dose-response modeling, and a single multiarm source would dominate dose contrast |
| Dose-duration pattern for body composition | Body-fat percentage; fat-free mass | Sparse rows with mixed post/change reporting and exercise-setting differences | Descriptive evidence map only | Mixed reporting basis, exercise context, product boundaries, and shared-control dose arms limit interpretability |
| Dose-duration pattern for strength outcomes | Bench-press 1RM; leg-press 1RM | Four rows for each strength outcome, with mixed post/change reporting and exercise context | Descriptive evidence map only | Performance outcomes are highly setting dependent, and the available dose contrast is not separable from training context and reporting basis |
| Dose context for symptom and sexual-function outcomes | AMS domains; AMS total; DISF total | Sparse rows, multiarm or coformulation boundaries, and symptom-score rather than hormone endpoints | Descriptive context only | These constructs are not interpretable as hormone dose-response outcomes |
| Funnel plot, publication-bias, and trial sequential analysis for dose contexts | All dose-duration questions | All relevant outcomes had small numbers of studies | Not performed | Conventional thresholds and the descriptive purpose of these questions did not support formal modeling |

#### Table S6B. Subgroup evidence map.

| **Dimension** | **Outcomes considered** | **Presentation** | **Rationale** |
| --- | --- | --- | --- |
| Exercise or athlete setting | Total testosterone; free testosterone; body-fat percentage; bench-press 1RM; leg-press 1RM | Described through sensitivity and interpretation rather than formal subgroup pooling | Exercise status and training cointerventions are confounded with intervention formulation and outcome type |
| Benign prostatic hyperplasia population | Total testosterone; free testosterone | Deletion sensitivity for the main testosterone-axis outcomes | The BPH trial is clinically distinct and better handled as a boundary sensitivity analysis than as a formal subgroup |
| Product or formulation boundary | Total testosterone; free testosterone; secondary and supplementary outcomes | Deletion sensitivity and narrative interpretation | Extract composition, coformulation status, and product family were heterogeneous and not consistently separable |
| Dose category | Total testosterone; free testosterone; selected supplementary outcomes | Descriptive dose context only | Dose cutpoints would be post hoc and unstable with the available study distribution |
| Duration category | Total testosterone; free testosterone; selected supplementary outcomes | Descriptive duration context only | Duration cutpoints would be post hoc and confounded by study population and formulation |
| Risk-of-bias or data-handling boundary | Main and supplementary outcomes | Sensitivity table or interpretation where relevant | These domains were better represented as sensitivity or data-handling checks than as inferential subgroups |

### Table S7. Reporting-basis and data-handling sensitivity rationale.

This table summarizes data-handling and reporting-basis considerations that informed interpretation of the quantitative results.

| **Issue** | **Affected outcomes or rows** | **Current handling** | **Interpretation note** |
| --- | --- | --- | --- |
| Mixed post/change reporting basis | Body-fat percentage; bench-press 1RM; leg-press 1RM; selected testosterone rows | Retained in supplementary presentation with reporting-basis caveats | Mixed basis limits direct comparability and supports interpreting these as supplementary findings |
| SEM-to-SD reconstruction | Guo 2018 total testosterone and any affected extracted rows | SDs reconstructed where required; deletion sensitivity available for total testosterone | Reconstruction did not create a new clinical claim and should be interpreted as a data-recovery step |
| Unit correction or source-label anomaly | Prolactin; fat-free mass; selected hormone-unit rows | Source labels and unit harmonization were checked before synthesis | These issues limit clean absolute-unit claims but do not alter the standardized main testosterone-axis interpretation |
| Figure reconstruction or digitized data | AMS outcomes and selected reconstructed rows | Retained in secondary analyses with caution | Reconstructed values support transparency but should not be over-weighted as primary evidence |
| Shared-control or multiarm structure | Hausenblas AMS rows; Rao 2020 calisthenics dose arms; other multiarm rows | One prespecified main comparison was retained where needed to avoid duplicate shared-control counting | Multiarm dose evidence was not treated as independent dose-response evidence |
| Dose-arm selection boundary | Rao 2020 and other dose-arm rows | Main analyses used a selected comparison; wider dose context was summarized descriptively | Dose-arm context should not be interpreted as a confirmatory dose-response analysis |
| Coformulation or product boundary | DISF total; prolactin; product-boundary rows | Retained as secondary, supplementary, or sensitivity context rather than main testosterone-axis evidence | Coformulations and mixed products constrain causal attribution to fenugreek alone |
| Free-testosterone unit/source trace | Poole 2010; Rao 2020 BPH | Preserved as a source-trace caveat in interpretation | These caveats reinforce caution when interpreting free testosterone |
| Hormone subset denominator | Rao 2020 calisthenics | Hormone-analysis denominators were separated from physical-performance denominators | Outcome-family denominators should not be mixed in dose or performance interpretations |
| Selected sensitivity plotting | Main testosterone-axis deletion sets and data-handling sensitivities | Summarized in tables rather than supplementary figures | Tables were preferred because small study numbers and multiple caveats would make figure interpretation fragile |

## Abbreviations

AMS, Aging Males’ Symptoms; BPH, benign prostatic hyperplasia; CI, confidence interval; DISF, Derogatis Interview for Sexual Function; GIV, generic inverse variance; ICTRP, International Clinical Trials Registry Platform; MD, mean difference; RCT, randomized controlled trial; REML, restricted maximum likelihood; SD, standard deviation; SEM, standard error of the mean; SHBG, sex hormone-binding globulin; TSA, trial sequential analysis; WHO, World Health Organization; 1RM, one-repetition maximum.
